# Immunohistochemical Expression of MSH2 and MSH6 Mismatch Repair Proteins and Their Pathological Correlations in Canine Primary Pulmonary Carcinoma

**DOI:** 10.3390/ani16172726

**Published:** 2026-09-02

**Authors:** Alexandra Morar, Corina Toma, Dragoș Hodor, Claudiu Gal, Ibrahima Mamadou Sall, Marian Taulescu, Cornel Cătoi

**Affiliations:** 1 Department of Veterinary Pathology, Faculty of Veterinary Medicine, University of Agricultural Sciences and Veterinary Medicine Cluj-Napoca, 400372 Cluj-Napoca, Romania; corina.toma@usamvcluj.ro (C.T.); dragos.hodor@usamvcluj.ro (D.H.); gal.claudiu@gmail.com (C.G.); ibrahima.sall@student.usamvcluj.ro (I.M.S.); marian.taulescu@usamvcluj.ro (M.T.); cornel.catoi@usamvcluj.ro (C.C.); 2 Department of Veterinary Pathology, Synevovet, Industriilor 25, 077040 Chiajna, Romania

**Keywords:** canine PPC, mismatch repair, MSH2, MSH6, immunohistochemistry, veterinary oncology

## Abstract

DNA mismatch repair (MMR) proteins have been investigated as biomarkers in human oncology, but their role in canine PPCs has not been characterized. Immunohistochemistry for MCK, TTF-1, MSH2, and MSH6 was performed and evaluated in relation to clinicopathological parameters. Affected dogs had a median age of 10.6 years. Histologically, 16 tumors were classified as adenocarcinoma and one as adenosquamous carcinoma, with papillary architecture predominating. MCK and TTF-1 immunoreactivity was detected in all cases, supporting epithelial and pulmonary differentiation when considered with histopathological and available clinical findings. Absent MSH2 immunoreactivity was observed in 17.6% of cases, whereas absent MSH6 immunoreactivity was observed in 11.8%. MSH2 expression showed a significant inverse association with tumor volume (*p* = 0.006), whereas no significant associations were identified between MSH6 expression and the evaluated clinicopathological parameters. As only MSH2 and MSH6 were assessed and no microsatellite instability or molecular analyses were performed, the observed abnormalities cannot be interpreted as definitive evidence of functional MMR deficiency. These findings provide preliminary data on MSH2 and MSH6 expression in canine PPCs and support further investigation using comprehensive MMR assessment and molecular approaches.

## Simple Summary

In human medicine, abnormalities in the DNA mismatch repair (MMR) system have been associated with several cancers; however, MMR protein expression in canine primary pulmonary carcinomas (PPCs) remains uncharacterized. In this study, 17 canine PPCs were evaluated using clinical information, gross and microscopic examination, and immunohistochemistry. The expression of two MMR proteins, MSH2 and MSH6, was investigated and showed variable staining among tumors, with absent immunoreactivity observed in a subset of cases. Reduced MSH2 expression was significantly associated with larger tumor size. These findings provide the first description of MSH2 and MSH6 immunohistochemical expression in canine PPCs and provide preliminary data for future studies investigating the potential relevance of MMR proteins in canine pulmonary tumors.

## 1. Introduction

Canine primary pulmonary carcinoma (PPC) is an uncommon primary pulmonary neoplasm that predominantly affects older dogs [1,2,3,4,5]. Among primary pulmonary tumors, adenocarcinoma (ADC) is the most frequently reported histological subtype [1,2,6]. Although the histopathological features of canine PPC have been well described, its molecular characteristics remain poorly understood. In particular, little is known about the potential involvement of DNA repair pathways in canine pulmonary tumors.

The DNA mismatch repair (MMR) system is a highly conserved DNA repair pathway responsible for identifying and correcting replication-associated errors, including base-base mismatches and insertion-deletion loops, thereby preserving genomic stability. In human oncology, immunohistochemical evaluation of MMR protein expression is an established approach for identifying tumors with deficient mismatch repair (dMMR). Among the MMR proteins, MutS homolog 2 (MSH2) and MutS homolog 6 (MSH6) form the MutSα heterodimer, which plays a critical role in mismatch recognition and initiation of DNA repair [7,8]. Loss of MMR function may result in the accumulation of replication errors and microsatellite instability (MSI), a molecular phenotype associated with several human cancers [9].

Germline alterations in MMR genes cause Lynch syndrome, a hereditary cancer predisposition syndrome mainly associated with colorectal and endometrial carcinomas, although other tumor types may also be affected [10,11,12]. Although *MLH1, PMS2, MSH2*, and *MSH6* are all relevant to Lynch syndrome and MMR deficiency, the present study specifically focused on MSH2 and MSH6 because they form the functionally related MutSα heterodimer involved in mismatch recognition. The aim was therefore not to provide a comprehensive assessment of the MMR protein panel, but to investigate this specific component of the MMR pathway.

Although gastrointestinal tumors represent the classical tumor context for Lynch syndrome, alterations in the MMR pathway have also been investigated in human lung cancer. MMR deficiency appears to be uncommon in lung cancer, but MMR-deficient and MSI-high lung tumors have been documented, including cases involving *MSH2/MSH6* alterations [13,14]. In addition, MSH2 and MSH6 expression has been investigated in relation to clinicopathological features of human lung cancer [15]. These findings suggest that, although MMR deficiency is not a major feature of most lung cancers, its potential involvement in pulmonary neoplasia warrants further investigation.

In veterinary medicine, the contribution of MMR pathway alterations to spontaneous neoplasms remains insufficiently characterized. Although MMR protein expression has been investigated in selected canine tumor types [16,17,18], the expression of MSH2 and MSH6 in canine PPC has not yet been reported. To the authors’ knowledge, no previous study has investigated the expression of MSH2 and MSH6 in canine PPC. Therefore, this study aimed to characterize the immunohistochemical expression of MSH2 and MSH6 in canine PPC and to evaluate their associations with relevant pathological and clinicopathological features.

## 2. Materials and Methods

### 2.1. Case Selection

A retrospective search of the archives of the Department of Veterinary Pathology, University of Agricultural Sciences and Veterinary Medicine Cluj-Napoca, Romania, and the Synevovet Laboratory, Bucharest, Romania, was performed to identify canine PPC cases diagnosed between 2020 and 2025. The following inclusion criteria were applied: (i) availability of formalin-fixed, paraffin-embedded tissue suitable for histological and immunohistochemical evaluation; (ii) diagnosis of PPC supported by histological features and immunohistochemical evaluation of multi-cytokeratin (MCK) and thyroid transcription factor 1 (TTF-1); (iii) absence of a documented extrapulmonary primary carcinoma based on the available clinical and pathological information; and (iv) availability of sufficient clinicopathological information, including age, sex, breed, and tumor size. Cases of metastatic pulmonary epithelial tumors, primary pulmonary non-epithelial neoplasms, and tumors with extensive necrosis precluding adequate histological or immunohistochemical evaluation were excluded.

A total of 17 cases met the inclusion criteria and were included in the study. Five cases were obtained during routine necropsy examinations, in which the absence of an extrapulmonary primary tumor was assessed during post-mortem examination. Twelve cases were submitted as biopsy specimens (lung lobectomy); for these cases, classification as primary pulmonary tumors was based on the available clinical history and diagnostic information, together with the histopathological findings and immunohistochemical evaluation. Cases with evidence or a documented history of an extrapulmonary primary carcinoma were excluded. Draining lymph nodes were included when available. Gross, histopathological, and clinical data were retrieved from institutional databases and medical records.

### 2.2. Histopathological Examination

Formalin-fixed, paraffin-embedded tissue samples were sectioned at 2–3 μm. The first section from each pulmonary tumor tissue block was stained with hematoxylin and eosin (H&E) for histopathological evaluation, while the subsequent four sections were used for immunohistochemical analyses. One tissue section was obtained from each available draining lymph node. All cases were evaluated using an Olympus BX41 light microscope (Olympus, Tokyo, Japan). Representative photomicrographs were acquired using an Olympus UC30 digital camera (Olympus, Tokyo, Japan) coupled with Stream Basic software, version 1.5.1.

Histological classification of pulmonary tumors was performed according to previously published criteria [5]. Mitotic activity was assessed manually by light microscopy using an Olympus BX41 microscope equipped with WHN10×/22 eyepieces and a 40× objective providing a total magnification of 400×. The field diameter was calculated as the eyepiece field number divided by the objective magnification (22/40 = 0.55 mm). The area of a high-power field was subsequently calculated using the formula A = π r^2^, where r is half of the field diameter, resulting in A = 0.2376 mm^2^. Accordingly, 10 high-power fields, corresponded to a total examined area of 2.37 mm^2^ [19]. Areas with the highest mitotic activity were selected, while regions of necrosis, desmoplasia, and inflammation were excluded from evaluation. Additional histological features, including vascular invasion, intratumoral necrosis, and inflammatory infiltrate, were qualitatively assessed.

### 2.3. Immunohistochemistry

Immunohistochemistry was performed for MCK, TTF-1, MSH2, and MSH6 (Table 1) in all canine pulmonary tumor samples using automated or manual staining procedures according to the antibody-specific protocols described below.

Immunostaining for MCK and TTF-1 was performed using an automated Leica BOND-Max system (Leica Biosystems, Melbourne, Australia; BOND-Max series M2 12154), with primary antibodies against MCK (clone AE1/AE3, catalog no. PA0909-U) and TTF-1 (clone SPT24, catalog no. PA0364) and BOND Polymer Refine Detection (Leica Biosystems, Melbourne, Australia, catalog no. NC0318637). Antigen retrieval and staining conditions were applied according to the manufacturer’s instructions. Normal and peritumoral lung tissues served as positive controls, whereas negative controls were obtained by replacing the primary antibody with non-immune serum of the corresponding species. MCK and TTF-1 immunoreactivity were evaluated qualitatively and recorded as present or absent, based on cytoplasmic and nuclear staining of neoplastic cells, respectively. Staining intensity and the proportion or distribution of immunoreactive tumor cells were not assessed.

Immunohistochemical detection of MSH2 and MSH6 was performed manually. Sections were deparaffinized in xylene and rehydrated through graded alcohols. Heat-induced epitope retrieval was performed using ethylenediaminetetraacetic acid (EDTA) buffer (pH 9.0) (EnVision FLEX Target Retrieval Solution, High pH, Dako, Glostrup, Denmark) for 20 min. Endogenous peroxidase activity was blocked using hydrogen peroxide, followed by protein blocking. Sections were incubated overnight at 4 °C with the ready-to-use primary antibodies against MSH2 (clone 79H11; catalog no: PA0989) and MSH6 (clone EP49, catalog no: PA0990), without further dilution. Immunoreactivity was visualized using the EnVision FLEX polymer-based detection system (Dako, Glostrup, Denmark) with 3,3′-diaminobenzidine (DAB) (Ventana Medical Systems, Tucson, AZ, USA) as the chromogen for 1 min, followed by counterstaining with Mayer’s hematoxylin.

For MSH2 and MSH6, lymph node cells, stromal cells, and lymphocytes within bronchus-associated lymphoid tissue (BALT) in normal and peritumoral lung tissues served as internal positive controls. Negative controls were prepared by omitting the primary antibody and replacing it with non-immune serum of the corresponding species.

MSH2 and MSH6 immunohistochemical expression was evaluated by manually counting all immunostained and non-immunostained tumor cell nuclei in 10 high-power fields (HPFs). The percentage of positive tumor nuclei was calculated and categorized according to the following staining proportion score: 0, 0%; 1, <10%; 2, 10–50%; 3, 51–80%; and 4, >80% positive tumor nuclei. Nuclear staining intensity (SI) was assessed in one representative HPF per case and scored as follows: 0, no detectable staining; 1, weak staining; 2, moderate staining; and 3, strong staining. Staining intensity was independently evaluated by two veterinary pathologists and results were compared for concordance.

An immunoreactive score (IRS), ranging from 0 to 12, was calculated for each case by multiplying the staining proportion score by the staining intensity score. IRS values were categorized as negative (0–1), weak (2–3), moderate (4–7), or strong (8–12) expression [20].

Complete loss of MSH2 or MSH6 expression was considered the absence of nuclear staining in neoplastic cells (0% positive tumor nuclei), with preserved nuclear staining in internal positive controls within the same tissue section.

### 2.4. In Silico Sequence Homology Analysis

To assess the predicted cross-species suitability of the anti-human MSH2 (clone 79H11) and MSH6 (clone EP49) antibodies, canonical human and canine protein sequences were retrieved from the NCBI Protein database and compared using Protein BLAST (NCBI BLAST+ v2.15.0)and Clustal Omega (EMBL-EBI, version 1.2.4) [21,22]. Human MSH2 (P43246) and MSH6 (P52701) were compared with their canine counterparts (A0A8I3MVG3 and A0A8I3RSQ0, respectively). The manufacturer-reported MSH2 immunogen (amino acids 1–304) showed 94.1% amino acid identity, while full-length MSH6 showed 92.8% identity between human and canine sequences. These high levels of sequence conservation supported the predicted cross-species reactivity of both antibodies in canine tissues.

### 2.5. Statistical Analysis

Statistical analysis was performed using IBM SPSS Statistics version 32.0.0 (IBM Corp., Armonk, NY, USA). Descriptive statistics were calculated for all investigated variables. The Shapiro–Wilk test was used to assess data distribution. Due to the limited sample size and the non-normal distribution of the study variables, all statistical analyses were considered exploratory, and non-parametric methods were applied throughout. Tumor volume was calculated from the three macroscopic tumor dimensions (length, width, and height) using the ellipsoid formula: $V=\frac{\pi }{6}\times L\times W\times H$, which is considered a standard method for estimating the volume of solid tumor masses [23].

Associations between MSH2 and MSH6 immunoreactive scores, as well as their associations with tumor volume and mitotic index, were explored using Spearman’s rank correlation coefficient. Exact *p*-values are reported throughout the manuscript. Statistical significance was defined as *p* < 0.05.

## 3. Results

### 3.1. Epidemiological and Clinical Characteristics

Among the 17 cases included in this study, primary pulmonary tumors were most identified in mixed-breed dogs (7/17; 41.2%), followed by Bichon-type dogs (4/17; 23.5%). Single cases were identified in a Beagle, Bull Terrier, English Cocker Spaniel, Bernese Mountain Dog, Pug, and West Highland White Terrier (1/17 each; 5.9%).

Most cases occurred in females (13/17; 76.5%), including 4/13 intact females (30.8%) and 9/13 spayed females (69.2%). Among males, 3/4 were intact (75.0%) and one was neutered (25.0%).

The median age at diagnosis was 10.6 years (range: 6–17 years), with the highest number of cases occurring between 10 and 11 years of age (7/17; 41.2%).

### 3.2. Gross Findings

**Figure 1:**
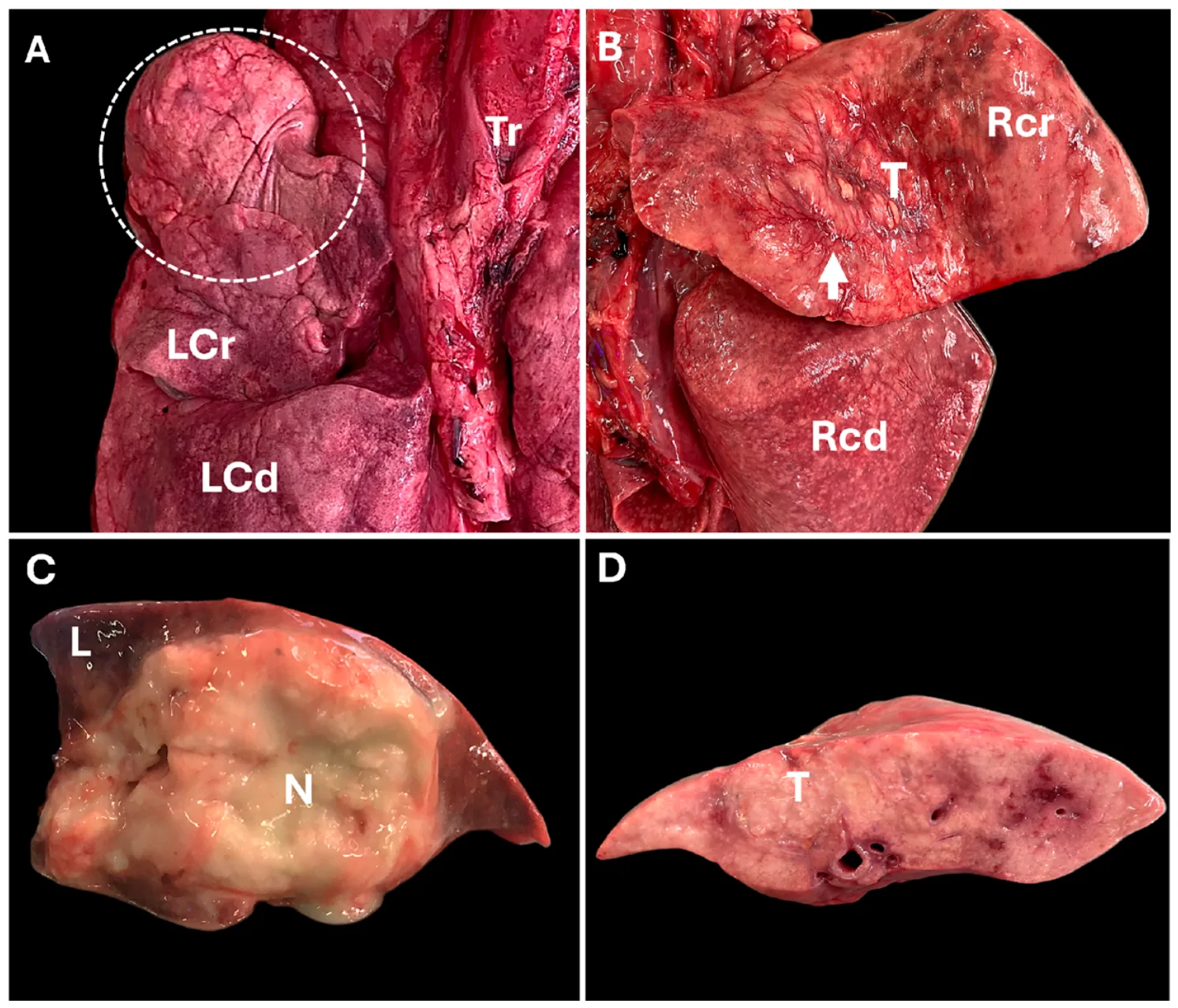
Gross features of canine primary pulmonary carcinomas. (**A**) Well-demarcated nodular mass (dashed circle) localized at the periphery of the left cranial lung lobe. (**B**) Poorly demarcated, infiltrative tumor (arrow) involving the right cranial lung lobe. (**C**) Cut section of a well-demarcated pulmonary mass. The lesion is predominantly composed of solid white-to-tan tissue with a locally extensive central area of necrosis. A rim of compressed, atelectatic, non-neoplastic pulmonary parenchyma surrounds the neoplasm. (**D**) Cut section of a pulmonary mass exhibiting an infiltrative growth pattern with poorly defined margins. The neoplastic tissue appears heterogeneous, with multifocal small pink-to-red areas compatible with intratumoral hemorrhage. Abbreviations: Tr, trachea; LCr, left cranial lung lobe; LCd, left caudal lung lobe; RCr, right cranial lung lobe; RCd, right caudal lung lobe; T, tumor; N, necrosis; L, non-neoplastic lung parenchyma.

Macroscopically (Figure 1A), most tumors were well-demarcated (11/17; 64.7%), whereas 6/17 (35.3%) exhibited poorly defined margins (Figure 1B). On cut section (Figure 1C,D), the masses were heterogeneous, white-to-tan in color with multifocal yellow areas of prominent necrosis in 7/17 cases (41.2%) and hemorrhage, occasionally forming cavitary changes. Tumor consistency was predominantly firm (9/17; 52.9%), whereas 8/17 tumors (47.1%) were classified as soft. Tumor size ranged from 2.2 × 1.8 to 12.5 × 11 cm (Table A1). Macroscopic evaluation of draining lymph nodes was available in 8/17 cases (47.1%). None of the examined lymph nodes showed gross evidence of metastatic involvement.

### 3.3. Histopathological Findings

**Figure 2:**
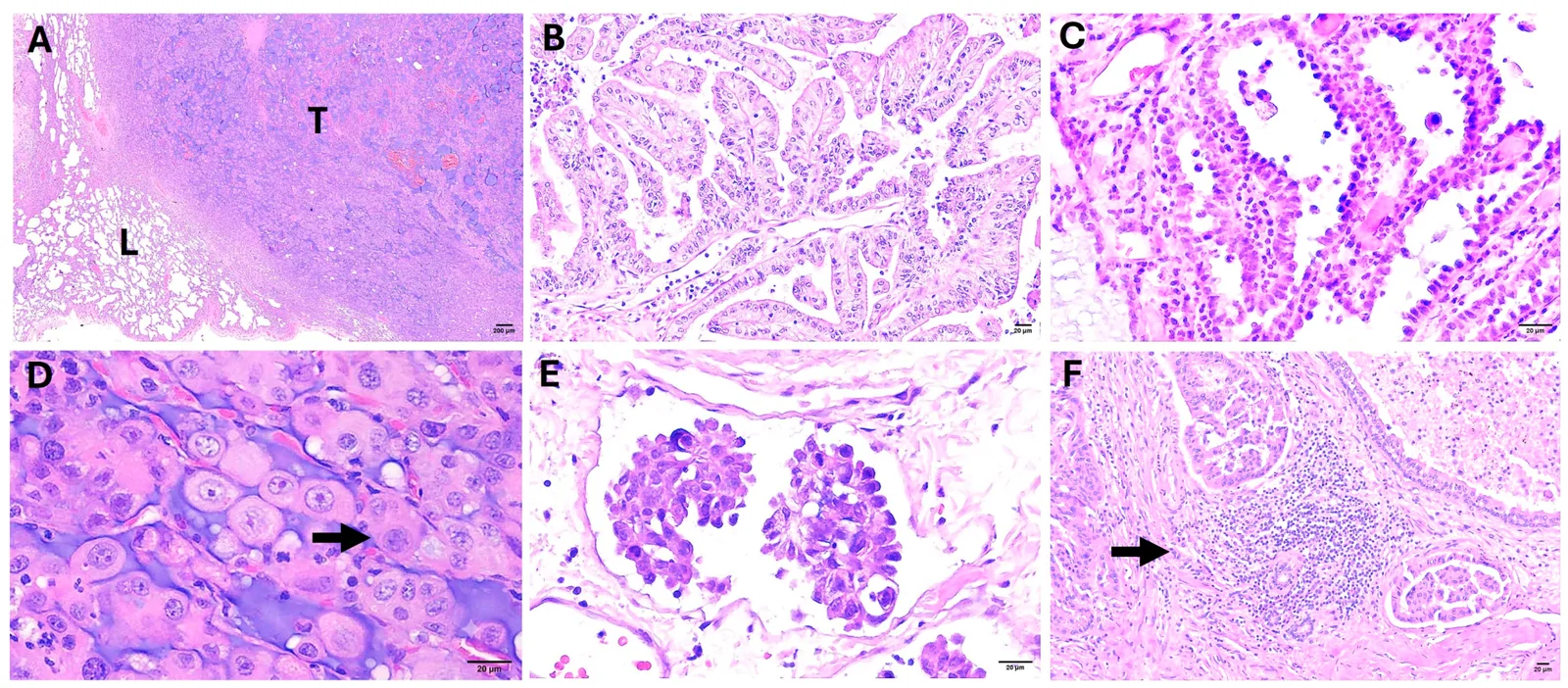
Histological features of primary pulmonary carcinomas. (**A**) Adenocarcinoma compressing and infiltrating the adjacent non-neoplastic pulmonary parenchyma. (**B**) Adenocarcinoma exhibiting a papillary pattern. (**C**) Adenocarcinoma exhibiting a lepidic pattern. (**D**) Adenocarcinoma with squamous differentiation (arrow). (**E**) Lymphatic vessel containing neoplastic emboli. (**F**) Neoplastic stroma showing multifocal infiltration by mononuclear inflammatory cells (arrow). Abbreviations: T, tumor; L, non-neoplastic lung parenchyma.

Canine PPC (Figure 2A) was histologically confirmed in all cases (17/17; 100%) (Table A1). Neoplasms were unencapsulated and mostly infiltrative, effacing and expanding the pulmonary parenchyma. Tumors were composed of neoplastic epithelial cells arranged predominantly in acinar and papillary patterns, with occasional solid areas, supported by a moderate fibrovascular stroma. In all cases, the neoplastic stroma was infiltrated by lymphocytes, with fewer macrophages and neutrophils. Neoplastic cells were variably flattened, cuboidal, or columnar, with distinct cell borders, moderate eosinophilic cytoplasm, and round to oval nuclei containing finely granular chromatin and one or multiple nucleoli. Anisocytosis and anisokaryosis were predominantly moderate. Mitotic activity ranged from 9 to 32 mitoses per 2.37 mm^2^ (Table A1).

Based on the predominant growth pattern, ADC, papillary type (Figure 2B) was most frequently diagnosed (15/17; 88.2%), followed by lepidic (Figure 2C) (1/17; 5.9%) and pulmonary adenosquamous carcinoma (ADS) (Figure 2D) (1/17; 5.9%).

Common histopathological features included multifocal to locally extensive necrosis, vascular invasion (Figure 2E), and the presence of inflammatory infiltrate in the neoplastic stroma (Figure 2F).

Regional lymph nodes were available for histological evaluation in 3/17 cases, represented by ipsilateral tracheobronchial lymph nodes, and no evidence of metastatic involvement was identified in any of the examined lymph nodes. Mild to moderate lymphoid hyperplasia was observed in all samples.

### 3.4. Immunohistochemical Findings

**Figure 3:**
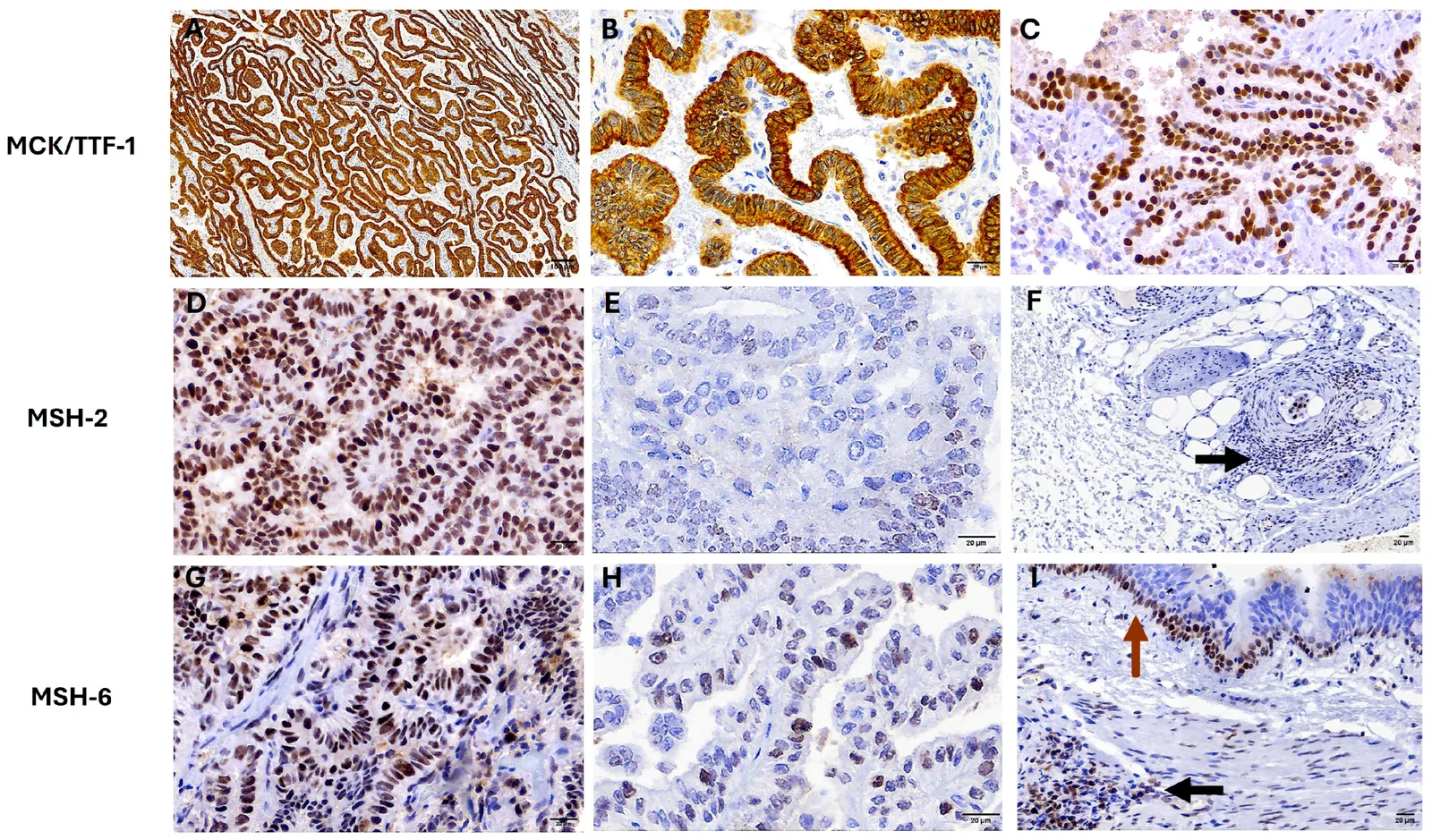
Immunohistochemical expression of MCK, TTF-1, MSH2, and MSH6 in canine primary pulmonary carcinomas. (**A**,**B**) Diffuse and intense cytoplasmic immunolabeling for MCK in neoplastic epithelial cells; (**C**) diffuse and intense nuclear immunolabeling for TTF-1 in neoplastic epithelial cells (**D**) MSH2 immunoexpression showing diffuse and strong nuclear immunolabeling; (**E**) MSH2 immunoexpression showing moderate nuclear immunolabeling with a low percentage of positive nuclei; (**F**) strong nuclear immunoreactivity for MSH2 in lymphocytes within BALT (arrow), serving as internal positive controls; (**G**) MSH6 immunoexpression showing diffuse and strong nuclear immunolabeling; (**H**) MSH6 immunoexpression showing moderate nuclear immunolabeling with a lower percentage of positive nuclei; (**I**) strong nuclear immunoreactivity for MSH6 in lymphocytes within BALT (black arrow), and in basal bronchiolar epithelium (brown arrow), serving as internal positive controls.

Immunohistochemically, all neoplastic cells (17/17; 100%) exhibited diffuse, strong cytoplasmic immunoreactivity for MCK (Figure 3A,B), confirming the epithelial origin of the tumors (Table A1). TTF-1 immunoreactivity was detected as nuclear labeling in all tumors (17/17; 100%) (Figure 3C); however, staining intensity was variable both among cases and within individual neoplasms, ranging from strong and diffuse to moderate, heterogeneous, and occasionally mild labeling (Table A1). TTF-1 expression was restricted to the neoplastic epithelial cell population, whereas the neoplastic stromal component was consistently negative. In normal lung tissue, nuclear TTF-1 immunoreactivity was observed in bronchiolar epithelial cells and type II pneumocytes, while alveolar septal mesenchymal cells were immunonegative.

In neoplastic epithelial cells, MSH2 nuclear immunoreactivity (Figure 3D,E) was present with intertumoral heterogeneity (Table A1). Loss of expression was observed in 3/17 cases (17.6%), whereas the remaining tumors (14/17; 82.4%) exhibited variable proportions of positively stained neoplastic cell nuclei, ranging from focal to near-diffuse immunolabeling (approximately 18–93%). Staining intensity ranged from absent to strong (scores 0–3), with absent intensity (score 0) observed in 3/17 cases (17.6%), weak intensity (score 1) in 3/17 cases (17.6%), moderate intensity (score 2) in 5/17 cases (29.4%), and strong intensity (score 3) in 6/17 cases (35.3%). The IRS was strong for 6/17 cases (35.3%), moderate for 6/17 cases (35.3%), weak for 2/17 cases (11.8%), and negative for 3/17 cases (17.6%).

MSH6 (Figure 3G,H) showed a comparable expression pattern, with loss of nuclear immunoreactivity in 2/17 cases (11.8%). The remaining tumors (15/17; 88.2%) demonstrated variable proportions of positively stained neoplastic cell nuclei (approximately 28.8–92.8%), with heterogeneous staining intensity ranging from score 0 to 3. Absent expression (score 0) was observed in 2/17 cases (11.8%), weak intensity (score 1) in 3/17 cases (17.6%), moderate intensity (score 2) in 5/17 cases (29.4%), and strong expression (score 3) in 7/17 cases (41.2%). The IRSs for MSH6 were strong in 6/17 cases (35.3%), moderate in 6/17 cases (35.3%), weak for 3/17 cases (17.6%), and negative for 2/17 cases (11.8%).

In the adjacent non-neoplastic pulmonary parenchyma, both MSH2 and MSH6 exhibited variable nuclear immunoreactivity in bronchiolar epithelial cells, alveolar epithelial cells, and lymphocytes, serving as internal positive controls (Figure 3F,I). Stromal mesenchymal elements were variably to minimally immunolabeled. Appropriate nuclear staining of internal positive controls was observed in all cases, including those in which neoplastic cells showed no detectable nuclear immunoreactivity (IRS = 0).

### 3.5. Statistical Analyses

**Figure 4:**
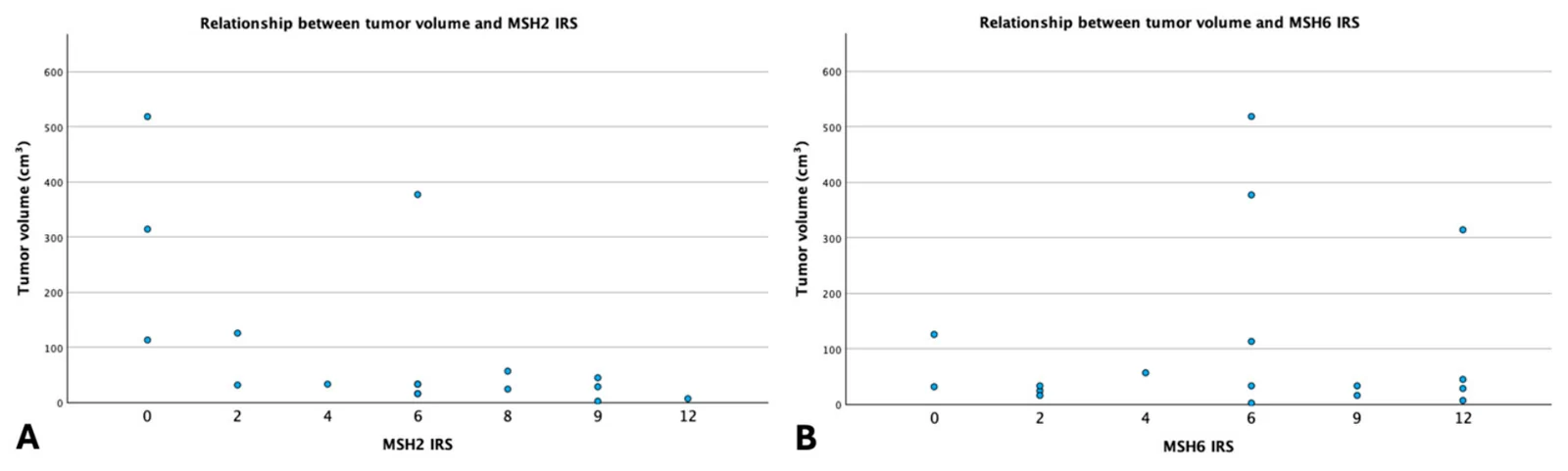
Scatterplots illustrating the relationship between tumor volume and MSH2 and MSH6 immunoreactive scores (IRS) in canine primary pulmonary carcinomas. (**A**) Tumor volume versus MSH2 IRS. (**B**) Tumor volume versus MSH6 IRS. The x-axis represents the immunoreactive score (IRS), and the y-axis represents tumor volume (cm^3^). Each point represents an individual tumor.

Spearman’s rank correlation analysis revealed no significant correlation between MSH2 and MSH6 immunoreactive scores (IRS) (ρ = 0.317, *p* = 0.215). Neither MSH2 nor MSH6 IRS was significantly correlated with the mitotic index (MSH2: ρ = −0.049, *p* = 0.853; MSH6: ρ = −0.377, *p* = 0.136). Tumor volume was not significantly correlated with the mitotic index (ρ = 0.201, *p* = 0.439) or MSH6 IRS (ρ = −0.041, *p* = 0.876). In contrast, MSH2 IRS was inversely correlated with tumor volume (ρ = −0.634, *p* = 0.006), with lower MSH2 expression observed in larger tumors (Figure 4).

## 4. Discussion

Canine primary pulmonary carcinoma (PPC) is an uncommon neoplasm, and the available veterinary literature consists largely of small retrospective case series describing its clinical and morphological features [1,2,24,25,26]. In the present study, the median age at diagnosis was 10.6 years, consistent with previous reports indicating that PPC predominantly affects older dogs [2,24]. Given the limited number of cases, the observed distributions of breed and sex should be interpreted cautiously and should not be considered indicative of breed or sex predisposition.

The gross and histopathological findings were broadly consistent with previously described canine PPCs [2,24,27]. Regional lymph node metastases were not identified in the available samples; however, lymph node assessment was limited and complete staging information was not available for all cases. Therefore, the absence of confirmed nodal metastasis should be interpreted cautiously.

Immunohistochemical findings supported the epithelial and pulmonary phenotype of the tumors, with MCK and TTF-1 immunoreactivity detected in all cases. These findings, considered together with the histopathological features and available clinical information, supported the classification of the lesions as primary pulmonary carcinomas [19,28].

The major objective of this study was to characterize MSH2 and MSH6 immunohistochemical expression in canine PPC. The MMR system is an evolutionarily conserved DNA repair pathway involved in correcting replication-associated errors. In human oncology, MMR protein immunohistochemistry is routinely used to assess MMR protein expression and to identify tumors that may warrant further evaluation for dMMR [9,29,30]. MSH2 and MSH6 form the MutSα complex, which participates in mismatch recognition and initiation of DNA repair [7]. Although MMR deficiency is particularly relevant in colorectal and endometrial carcinomas, alterations in MMR proteins have also been investigated in human lung cancer, where dMMR appears to be uncommon [13,14]. In veterinary medicine, the contribution of MMR abnormalities to spontaneous tumor development remains poorly characterized. Previous studies have demonstrated that altered MMR protein immunoreactivity can occur in selected canine neoplasms, although its frequency varies among tumor types [16].

In the present study, most PPCs retained expression of both MSH2 and MSH6. Altered MSH2 immunoreactivity was identified in three tumors (17.6%), whereas altered MSH6 immunoreactivity occurred in two tumors (11.8%). These findings indicate that altered immunohistochemical expression of individual MMR proteins may occur in a subset of canine PPCs. However, the present findings do not establish functional MMR deficiency or disruption of the overall MMR pathway. Loss or reduction in immunohistochemical expression may result from biological mechanisms, pre-analytical or analytical factors, or antibody-related effects and therefore requires cautious interpretation [31]. In particular, none of the tumors demonstrated concurrent loss of both MSH2 and MSH6 expression. The biological significance of these discordant staining patterns cannot be determined from immunohistochemistry alone. Accordingly, the observed abnormalities should be interpreted as altered MSH2 or MSH6 protein expression rather than confirmed dMMR.

The predominance of retained MSH2 and MSH6 expression is broadly consistent with findings in human lung cancer, in which MMR deficiency is uncommon [13,14]. In contrast to tumor types such as colorectal carcinoma, where MMR deficiency represents an important molecular subtype, lung carcinogenesis is more commonly associated with alterations in other oncogenic pathways [4,32]. Therefore, the relatively low frequency of altered MSH2/MSH6 immunoreactivity observed in this study does not support MMR dysfunction as a predominant feature of canine PPC. However, given the limited number of cases and the restricted assessment of only two MMR proteins, this finding should be regarded as preliminary.

MSH2 immunohistochemical expression was inversely correlated with tumor volume. The biological relevance of this finding is unclear, although reduced MSH2 expression in larger tumors may reflect tumor heterogeneity or changes associated with disease progression. Given the small sample size and observational design, a causal relationship between MSH2 expression and tumor progression cannot be inferred. MSH6 expression was not associated with tumor volume or mitotic index. Further studies with larger sample sizes are needed to determine whether these findings represent biologically relevant variation or are influenced by technical and preanalytical factors.

Several limitations should be considered. The retrospective design and relatively small number of cases represent the principal limitations of this study. Furthermore, only MSH2 and MSH6 were evaluated by immunohistochemistry; therefore, a comprehensive assessment of MMR status was not possible. MLH1 and PMS2 expression, MSI status, and MMR gene alterations were not assessed. In addition, interpretation of immunohistochemical expression may be influenced by pre-analytical and analytical factors. Finally, the absence of metastatic disease in the available lymph nodes should be interpreted cautiously because complete staging information was not available for all cases.

Despite these limitations, this study provides the first characterization of MSH2 and MSH6 immunohistochemical expression in canine PPC. Most tumors retained expression of both proteins, whereas a subset demonstrated altered expression of individual MMR components. These findings provide preliminary evidence that MSH2 and MSH6 expression may vary among canine PPCs but do not establish functional MMR deficiency or broader MMR pathway disruption. Larger studies incorporating the complete MMR IHC panel, MSI assessment, and molecular characterization are required to determine the biological significance of MMR protein expression in canine pulmonary carcinomas.

## 5. Conclusions

This study provides the first characterization of MSH2 and MSH6 immunohistochemical expression in canine PPCs. The evaluated tumors showed consistent epithelial and pulmonary differentiation based on MCK and TTF-1 immunoreactivity and displayed morphological features comparable with previously reported canine PPCs. Most tumors retained nuclear expression of both MSH2 and MSH6, whereas a subset showed reduced or absent immunoreactivity for one of these proteins. These findings indicate that altered MSH2 or MSH6 expression occurs in a subset of canine PPCs, but do not establish functional MMR deficiency or disruption of the overall MMR pathway. However, the identification of tumors with reduced or absent expression of individual MMR proteins indicates that alterations in this pathway may occur in a subset of cases. The association between decreased MSH2 expression and increased tumor volume may indicate a relationship between MSH2 protein expression and tumor progression; however, the biological significance and direction of this association remain uncertain. Further studies incorporating larger case series, the complete MMR IHC panel, microsatellite instability assessment, and molecular analyses are required to determine the significance of MSH2 and MSH6 expression patterns in canine PPCs. These findings provide preliminary data for further investigation of DNA repair mechanisms in canine pulmonary carcinogenesis.

## Figures and Tables

**Table 1:** Primary antibodies and immunohistochemical conditions used for the evaluation of canine primary pulmonary carcinomas.

Marker	Manufacturer	Dilution	Clone	Antigen Retrieval
Multicytokeratin (MCK)	LeicaBiosystems, Melbourne, Australia	RTU	AE1/AE3	Enzymatic
Thyroid Transcription Factor-1 (TTF-1)	LeicaBiosystems, Melbourne, Australia	RTU	SPT24	Citrate buffer (pH6)
MutS protein homolog 2 (MSH2)	LeicaBiosystems, Melbourne, Australia	RTU	79H11	EDTA buffer (pH9)
MutS protein homolog 6 (MSH6)	LeicaBiosystems, Melbourne, Australia	RTU	EP49	EDTA buffer (pH9)

## Data Availability

The original contributions presented in this study are included in the article. Further inquiries can be directed at the corresponding author.

## Abbreviations

The following abbreviations are used in this manuscript:

ADC	Adenocarcinoma
ADS	Adenosquamos carcinoma
BALT	Bronchus-associated lymphoid tissue
DAB	3,3′-diaminobenzidine
dMMR	Deficient mismatch repair
DNA	Deoxyribonucleic acid
FFPE	Formalin-fixed, paraffin-embedded
FN	Field number
H&E	Hematoxylin and eosin
HPF	High-power field
IHC	Immunohistochemistry
Int.	Staining intensity
IRS	Immunoreactive score
L	Non-neoplastic lung parenchyma
LCd	Left caudal lung lobe
LCr	Left cranial lung lobe
MCK	Multi-cytokeratin
MI	Mitotic index
MLH-1	MutL protein homolog 1
MMR	Mismatch repair
MSH2	MutS homolog 2
MSH6	MutS homolog 6
MSI	Microsatellite instability
N	Necrosis
PMS-2	Postmeiotic segregation increased 2
PPC	Primary pulmonary carcinoma
Prop.	Proportion of positive tumor cells
RCd	Right caudal lung lobe
RCr	Right cranial lung lobe
SI	Staining intensity
T	Tumor
Tr	Trachea
TTF-1	Thyroid transcription factor-1

## Appendix A

**Table A1:** Tumor characteristics and immunohistochemical profiling of canine primary pulmonary carcinomas.

Case	Histological Diagnosis	Tumor Size (cm)	MI	MCK	TTF-1	MSH2 Prop. (Score)	MSH2 Int.	MSH2 IRS	MSH6 Prop. (Score)	MSH6 Int.	MSH6 IRS
1	ADC, papillary type	10 × 8 × 9	18	+	+	28.5% (2)	3	6	43.4% (2)	3	6
2	ADC, papillary type	6 × 5 × 1	10	+	+	18.8% (2)	3	6	78.7% (3)	3	9
3	ADC, lepidic type	10 × 8 × 7.5	12	+	+	0% (0)	0	0	83.2% (4)	3	12
4	ADC, papillary type	9 × 6 × 4	12	+	+	0% (0)	0	0	53.9% (3)	2	6
5	ADC, papillary type	6 × 3 × 3	32	+	+	58.8% (3)	3	9	88.6% (4)	3	12
6	ADC, papillary type	2.2 × 1.8 × 1	11	+	+	36.8% (3)	3	9	63.8% (3)	2	6
7	ADC, papillary type	12.5 × 11 × 7.2	11	+	+	0% (0)	0	0	45.4% (3)	2	6
8	ADC, papillary type	4.5 × 4 × 3.5	9	+	+	55.8% (3)	2	6	80.7% (3)	3	9
9	ADS	4 × 3.2 × 1	11	+	+	82.6% (4)	3	12	92.8% (4)	3	12
10	ADC, papillary type	6.2 × 5.5 × 2.5	16	+	+	74.9% (3)	3	9	91.6% (4)	3	12
11	ADC, papillary type	8 × 6 × 5	22	+	+	18.3% (2)	1	2	0% (0)	0	0
12	ADC, papillary type	6 × 5 × 2	18	+	+	36.3% (2)	1	2	0% (0)	0	0
13	ADC, papillary type	5.1 × 3 × 3	12	+	+	92.8%(4)	2	8	34.7% (2)	1	2
14	ADC, papillary type	4.8 × 2.5 × 2.5	17	+	+	76.5% (3)	2	6	36.4% (2)	1	2
15	ADC, papillary type	4.5 × 4 × 3.5	20	+	+	73.5% (3)	2	6	41.2% (2)	1	2
16	ADC, papillary type	7.2 × 5 × 3	12	+	+	88.3% (4)	2	8	28.8% (2)	2	4
17	ADC, papillary type	3.5 × 4 × 4.5	15	+	+	84.8% (4)	1	4	52.4% (3)	2	6

Abbreviations: ADC: Adenocarcinoma; ADS: Adenosquamous carcinoma; MI: Mitotic index (mitoses per 2.37 mm^2^); MCK: Multi-cytokeratin; TTF-1: Thyroid transcription factor-1; Prop.: Proportion of positive tumor cells (score 1–4 provided in parentheses); Int.: Staining intensity (score 0–3); IRS: Immunoreactive score (Intensity score × Proportion score).
